# Environmental pollutants increase the risks of acute exacerbation in patients with chronic airway disease

**DOI:** 10.3389/fpubh.2023.1215224

**Published:** 2023-10-30

**Authors:** Chien-Hong Chou, Yen-Fu Chen, Hung-Chueh Peng, Chung-Yu Chen, Bor-Wen Cheng

**Affiliations:** ^1^Department of Internal Medicine, National Taiwan University Hospital Yunlin Branch, Yunlin, Taiwan; ^2^Division of Pulmonary and Critical Care Medicine, Department of Internal Medicine, National Taiwan University Hospital and College of Medicine, National Taiwan University, Taipei, Taiwan; ^3^Department of Industrial Engineering and Management, National Yunlin University of Science and Technology, Yunlin, Taiwan

**Keywords:** air pollution, chronic airway disease, acute exacerbation, nitrogen dioxide, ozone

## Abstract

**Objective:**

Respiratory infections are a common cause of acute exacerbations in patients with chronic airway disease, however, environmental factors such as air pollution can also contribute to these exacerbations. The study aimed to determine the correlation between pollutant levels and exacerbation risks in areas exposed to environmental pollution sources.

**Methods:**

From 2015 to 2016, a total of 788 patients with chronic airway diseases were enrolled in a study. Their medical records, including hospital visits due to acute exacerbations of varying severity were analyzed. Additionally, data on daily pollutant levels from the Air Quality Monitoring Network from 2014 to 2016 was also collected and analyzed.

**Results:**

Patients with chronic airway disease and poor lung function (FEV1 < 50% or obstructive ventilatory defect) have a higher risk of severe acute exacerbations and are more likely to experience more than two severe acute exacerbations within a year. The study found that in areas exposed to environmental pollution sources, there is a significant correlation between NO_2_, O_3_, and humidity with the main causes of severe acute exacerbation. When the levels of NO_2_ were higher than 16.65 ppb, O_3_ higher than 35.65 ppb, or humidity higher than 76.95%, the risk of severe acute exacerbation in patients with chronic airway disease increased.

**Conclusion:**

Acute exacerbations of chronic airway disease can be triggered by both the underlying disease state and the presence of air pollution. Computer simulations and early warning systems should be developed to predict acute exacerbations of chronic airway disease based on dynamic changes in air pollution.

## Introduction

Chronic airway diseases, such as chronic obstructive pulmonary disease (COPD) and asthma, are characterized by chronic inflammation of the airways. Both conditions have common risk factors, such as tobacco smoking, exposure to air pollution, pulmonary infection, genetics, and aging. Smoking is the main cause of COPD (1), leading to increased respiratory symptoms and higher mortality rates due to rapid decline in lung function from long-term tobacco smoke exposure (2).

Exposure to high doses of pesticides, dusts, chemical agents, and fumes in environmental settings also contribute to the burden of COPD and asthma (3). Exposure to particulate matter (PM), such as PM_2.5_, is also a significant risk factor for COPD and asthma. Studies have shown that the concentration of PM_2.5_ in households with smokers is 10 times higher than in households without smokers (4). High concentrations of PM_2.5_ could increase the risk of hospitalization in COPD patients (5). Studies have also demonstrated a correlation between exposure to PM_2.5_ and respiratory infections (6). An increase of 10 μg/m^3^ of PM_2.5_ per cubic meter can increase the mortality rate of cardiopulmonary diseases by 6%. Conversely, a decrease of 10 μg/m^3^ in PM_2.5_ concentration can increase the average life expectancy of residents in an area by 0.61 years (7).

Asthma is a chronic lung disease that affects millions of people worldwide. The causes of asthma are varied and can include genetics, allergies, infections, or a combination of these factors. About 30% of asthmatic patients have an allergic constitution, with dust mites being the most common and important allergen in Taiwan (8). A recent integrated analysis study has shown that exposure to traffic-related air pollution during childhood, such as black carbon, NO_2_, PM_2.5_, and PM_10_, is statistically significantly positively correlated with the subsequent occurrence of asthma (9). Additionally, a large study involving over 600,000 participants across three European generations found that long-term exposure to environmental air pollutants, particularly PM_10_ is positively associated with the prevalence of asthma (10). The study found that a 10 mg/m^3^ increase in PM_10_ concentration resulted in a 12.8% increase in asthma prevalence, and the effect was particularly significant in individuals over the age of 50, with a history of smoking and lower educational levels.

Acute exacerbation of chronic airway diseases can lead to a decline in the quality of life of patients, and may result in complications such as sepsis or cardiopulmonary failure. In severe cases, it may even increase the risk of death and cause a heavy social and economic burden and medical expenditure. Current clinical treatments for chronic airway diseases mainly consist of inhaled bronchodilators and inhaled steroids, which can improve symptoms and reduce the risk of acute exacerbation (1). However, even with regular drug use, external environmental factors such as exposure to allergens, air pollutants, mold, haze, and cigarettes can still cause acute exacerbation. Respiratory tract infections are the most common cause of acute exacerbation, but about two-thirds of patients with acute exacerbation have no clear pathogenic bacteria.

Air pollution, particularly in urban areas, can have negative effects on individuals with pre-existing heart or lung conditions. Studies have shown a correlation between high levels of particulate matter in the air and increased incidence of COPD and asthma (11). However, more research is needed to fully understand the relationship between air pollution and respiratory health, including the effects of both short-term, high-peak exposures and long-term, low-level exposures (12). Numerous studies have linked air pollution to the development and exacerbation of airway diseases. However, identifying the specific pollutants or combinations that have the greatest impact on chronic airway diseases is crucial for targeted interventions. Investigating the susceptibility of certain populations, including those with pre-existing respiratory conditions, is also necessary. Further evaluation of mitigation strategies is required to effectively reduce air pollution and improve respiratory health. Therefore, a comprehensive research is required to gain a deeper understanding of how specific components of air pollution interact with other contributing factors in the development and exacerbation of airway diseases. The goal of this study is to investigate the relationship between intrinsic clinical conditions and extrinsic environmental factors (air pollution) and its impact on severe exacerbation of chronic respiratory diseases. The study will determine the correlation between air pollutant changes and acute exacerbations and use statistics to analyze the influence of intrinsic and extrinsic factors on exacerbation.

## Materials and methods

The study participants of this research are patients diagnosed with chronic airway diseases in a medical center in Yunlin County, Taiwan, from 2015 to 2016, to explore the intrinsic clinical factors (age, gender, diagnosis classification, smoking history, comorbidities, laboratory examination values, lung function, blood data) and extrinsic environmental factors (air pollution indicators, carbon monoxide, nitrogen dioxide, ozone, PM_10_, PM_2.5_, sulfur dioxide, humidity, daily maximum temperature, daily minimum temperature) of these patients and, respectively, with whether there is severe acute exacerbation, and the frequency of exacerbation in these 2 years.

### Research participation

The main purpose of this study is to investigate the impact of environmental factors on the long-term control of severe chronic airway disease patients. The study used the International Statistical Classification of Diseases and Related Health Problems 9th Revision (ICD−9) codes CM490−493, 496 and ICD−10 codes J42—J46, which are codes for COPD, Asthma and Asthma/COPD overlapping (ACO), to select patients with chronic airway diseases who were diagnosed during their visits to National Taiwan University Hospital Yunlin Branch in Douliou City, Yunlin County, Taiwan. Participants under 18 years old and those who did not continue to receive treatment after diagnosis would be excluded from the study.

### Data collection

This is a retrospective study, and it was approved by the Research Ethics Committee with approval number 201411019RINB. Data collection from medical records included basic information such as sex, age, smoking history, medical history (cardiovascular disease, chronic kidney disease, diabetes, tuberculosis, viral hepatitis, lung cancer, other malignant tumors, etc.), diagnosis of chronic respiratory disease (COPD, asthma, ACO), time of diagnosis, laboratory values [such as white blood cell count (WBC) and its differentiation, IgE, allergen screening], and date and events of acute exacerbation to emergency department (ED) or hospital admission.

The diagnosis of COPD is based on symptoms such as dyspnea, chronic cough, sputum production, a history of recurrent lower respiratory tract infections, and exposure to risk factors. Confirming the diagnosis requires spirometry with a forced vital capacity maneuver, which shows a post-bronchodilator FEV1/FVC ratio below 0.7. (1). In contrast, asthma diagnosis relies on characteristic symptom patterns and evidence of variable expiratory airflow limitation, which is confirmed through bronchodilator reversibility testing (13). Asthma-COPD Overlap Syndrome (ACOS) is characterized by persistent airflow limitation and combines features of both asthma and COPD. It is identified by the presence of shared characteristics from both conditions (14).

The lung function test uses the Master Screen Body (JAEGER, GERMANY) instrument and the bronchodilator test, with the medication used being Berotec N (Fenoterol) Metered Aerosol 100 mcg/puff, 200 puffs/btl, and the data collected mainly includes post-forced expiratory volume in one second (FEV1) and post-FEV1/forced vital capacity (FVC) values.

The environmental factor registration data archives historical information from the Executive Yuan's Air Quality Monitoring Network, specifically from the Douliou Station. This data encompasses a range of critical metrics, including the air pollution index (PSI), carbon monoxide (CO), nitrogen dioxide (NO_2_), ozone (O_3_), PM_10_, PM_2.5_, sulfur dioxide (SO_2_), humidity, daily high and low temperature.

Severe acute exacerbation of chronic airway disease is defined as a sudden and severe worsening of symptoms (e.g., coughing, wheezing, shortness of breath), which lead to visit emergency department or hospitalization.

### Data processing and statistical analysis

This study used SPSS 22 software to perform various statistical analyses. Participant number, percentage, mean, and standard deviation were used to describe the distribution of participants with chronic airway diseases during acute exacerbation.

Pearson's chi-square test was utilized to assess the impact of intrinsic factors, which are categorical variables encompassing age (≥70 years or below), gender (female or male), diagnosis (asthma, COPD, or ACO), smoking history (ex- and current or non-smokers), lung function (FEV1 ≥ 80%, 50% ≤ FEV1 < 80%, 30% ≤ FEV1 < 50%, FEV1 < 30%), comorbidities (presence or absence of diabetes mellitus, cardiovascular disease, and lung cancer), and blood data (blood eosinophil > 2 or ≤ 2%, neutrophil > 65 or ≤ 65%), with respect to their influence on the frequency and presence of severe exacerbations in chronic airway disease. Univariate analysis was initially employed to identify intrinsic factors that significantly affect acute exacerbations, followed by multivariate logistic regression analysis to identify the most significant independent factors.

The *T*-test was employed to assess continuous variables and ascertain whether extrinsic environmental factors, specifically daily pollution values comprising CO, NO_2_, O_3_, SO_2_, PM_10_, and humidity, exerted a significant influence on the occurrence of exacerbations. Subsequently, a multivariate logistic regression analysis was conducted to identify the primary extrinsic factors contributing to acute exacerbations in patients with chronic airway disease.

ANOVA was employed to investigate the average monthly concentrations of NO_2_, O_3_, and humidity. Pearson's chi-square analysis was performed to inspect the occurrences of events in the months when patients with chronic airway diseases necessitated emergency care or hospitalization due to acute exacerbation. Furthermore, a multivariate linear regression analysis was conducted to evaluate the impact of NO_2_, O_3_, and humidity on the occurrence of severe acute exacerbations in individuals with chronic airway diseases.

Receiver operating characteristic (ROC) curves were employed to pinpoint pollution thresholds most likely to trigger acute exacerbations in patients with chronic airway diseases. Statistical significance was determined using a threshold of *p*-values below 0.05.

## Results

### Basic characteristics of patients with chronic airway disease

This study collected a total of 4,678 participants of patients diagnosed with chronic airway diseases and excluded 3,890 participants of patients who were under 18 years old or who did not continue to seek medical treatment after diagnosis, resulting in a total of 788 participants.

There were a total of 558 patients (70.8%) with COPD, 175 patients (22.2%) with Asthma, and 55 patients (7.0%) with ACO. The average age was 68.4 years old, with 417 patients (52.9%) being over 70 years old and there were 580 male patients (73.6%). Of the chronic airway disease patients collected in this study, 211 (26.8%) had never smoked, 162 (20.6%) were currently smoking, 311 (39.5%) had quit smoking, 7 (0.9%) were exposed to passive smoke. In terms of lung function, 259 patients (32.9%) had a Post-FEV1% >80%, 261 patients (33.1%) had a Post-FEV1% between 50 and 80, 108 patients (13.7%) had a Post-FEV1% between 30 and 50%, and 13 patients (1.6%) had a post-FEV1% < 30%.

The most common comorbidities among chronic airway disease patients in this study were cardiovascular disease (CVD) (34.3%, *n* = 270), followed by chronic kidney disease (CKD) (17.6%, *n* = 139), diabetes mellitus (DM) (16.5%, *n* = 130), and lung cancer (3.6%, *n* = 28) (Table 1).

**Table 1:** The basic characteristics of patients with chronic respiratory diseases.

**Clinical data**	***N* (%)**
Age (mean ± standard deviation) (years old)	68.4 ± 14.2
≧70	417 (52.9)
< 70	371 (47.1)
**Gender**	
Male	580 (73.6)
Female	208 (26.4)
**Cigarrete smoking**	
None	211 (26.8)
Current	162 (20.6)
Quitted	311 (39.5)
Passive	7 (0.9)
Unreported	97 (12.3)
**Diagnosis**	
COPD	558 (70.8)
Asthma	175 (22.2)
ACO	55 (7.0)
**Lung function Post-FEV1%**	
FEV1 ≧ 80%	259 (32.9)
50% ≦ FEV1 < 80%	261 (33.1)
30% ≦ FEV1 < 50%	108 (13.7)
FEV1 < 30%	13 (1.6)
No report	147 (18.7)
**Comorbidities**	
Cardiovascular disease	270 (34.3)
Chronic kidney disease	139 (17.6)
Diabetes mellitus	130 (16.5)
Pulmonary tuberculosis	45 (5.7)
Virus hepatitis	37 (4.7)
Lung cancer	28 (3.6)
Other malignancy	43 (5.5)
Other disease	44 (5.6)
**Blood biomarkers**	**Mean** ±**standard deviation**
WBC (mm^3^)	10.14 ± 43.70
Neutrophil counts (μl)	6,031.63 ± 31,630.94
Neutrophil (%)	67.41 ± 13.60
Eosinophil counts (μl)	228.55 ± 1,025.11
Eosinophil (%)	2.48 ± 2.95
IgE (U/L)	267.79 ± 525.09
**Phadiotop**	(*n* = 158, %)
Positive	79 (50.0)
Dust mite	57 (36.1)
Cockroach, German	30 (19.0)
Fungus	13 (8.2)
Animal (mixes)	11 (7.0)
Food	9 (5.7)
Grass (mixes)	5 (3.2)

COPD, chronic obstructive pulmonary disease; ACO, asthma/COPD overlapping; FEV1, forced expiratory volume in one second.

### Blood biomarkers of patients with chronic airway diseases

In this study, several blood markers were collected from patients with chronic airway diseases. The mean value of white blood cells (WBC) was 10,140/mm^3^, the mean value of neutrophils was 6,031.63/μl, the mean percentage of neutrophils was 67.41%, the mean value of eosinophils was 228.55/μl, the mean percentage of eosinophils was 2.48%, and the mean value of IgE was 267.79 U/L. Additionally, 158 patients underwent allergy screening, with dust mite allergy being the most common at 36.1%, followed by German cockroach allergy at 19.0%, fungus allergy at 8.2%, animal fur allergy at 7.0%, food allergy at 5.7%, and grass pollen allergy at 3.2% (Table 1).

### Analysis of the intrinsic clinical factors that impact severe acute exacerbations in patients with chronic airway disease

The elderly group (70 years or older) had a higher likelihood of severe acute exacerbations of their chronic airway disease, with 39.09% experiencing such exacerbations (*p* < 0.001). Additionally, this group was found to be more likely to experience more than two exacerbations per year (21.10%, *p* = 0.004).

Male patients had a higher chance of experiencing severe acute exacerbations of their chronic airway disease, with 35.34% experiencing such exacerbations (*p* = 0.05). Furthermore, men were found to be more likely to have more than two severe acute exacerbations per year than women (19.14%, *p* = 0.03).

Patients with ACO had a higher likelihood of experiencing severe acute exacerbations (41.82%, *p* < 0.001) compared to other airway disease groups. Both COPD and ACO patients were more likely to experience two or more severe acute exacerbations per year (20.79 and 7.43%, respectively) compared to Asthma patients (*p* < 0.001).

Patients who have ever smoked or currently smoke had a correlation with severe acute exacerbation. The proportion of patients who have ever smoked or currently smoke and have severe acute exacerbation is higher at 36.58% (*p* = 0.02). The proportion of patients who have ever smoked or currently smoke and have two or more severe acute exacerbations per year is significantly higher at 19.87% (*p* = 0.024) than that of patients who have never smoked.

Patients with worse lung function (FEV1 between 30 and 50%) had a higher proportion of severe acute exacerbation (51.85%, *p* < 0.001). The proportion of patients with FEV1 < 30% and have two or more severe acute exacerbations per year is significantly higher at 30.77% (*p* < 0.001) than those with better lung function.

Patients with a history of diabetes have a higher likelihood of experiencing severe acute exacerbations (43.85%, *p* = 0.006). Patients with diabetes were found to be more likely to have two or more severe acute exacerbations per year (22.59%, *p* = 0.03). Similarly, patients with a history of cardiovascular disease were more likely to experience severe acute exacerbations (41.11%, *p* = 0.001) and have a correlation with the number of severe exacerbations per year, with those more likely to have two or more severe acute exacerbations per year (22.59%, *p* = 0.005). Additionally, patients with a history of lung cancer had a higher probability of severe acute exacerbation (57.14%, *p* = 0.007) and were also found to have a correlation with the number of severe exacerbations per year, with those more likely to have two or more severe acute exacerbations per year (39.29%, *p* = 0.002) (Table 2).

**Table 2:** The relationship between intrinsic clinical factors and severe acute exacerbation (AE) in chronic airway diseases.

	**Severe AE**				***X^2^***	**Events of severe AE in 1 year** ^**^				***X^2^***
	**No**		**Yes**			≦**1**		≧**2**		
	***n*** = **525**		***n*** = **263**			***n*** = **651**		***n*** = **137**		
**Age (** ***n*** **, %)**										
< 70 y/o	271	73.05%	100	26.95%	13.00^***^	322	86.79%	49	13.21%	8.521^**^
≧70 y/o	254	60.91%	163	39.09%		329	78.90%	88	21.10%	
**Gender (** ***n*** **, %)**										
Male	375	64.66%	205	35.34%	3.83^*^	469	80.86%	111	19.14%	4.697^*^
Female	150	72.12%	58	27.88%		182	87.50%	26	12.50%	
**Diagnosis (** ***n*** **, %)**										
COPD	355	63.62%	203	36.38%	15.81^***^	442	79.21%	116	20.79%	16.89^***^
Asthma	138	78.86%	37	21.14%		162	92.57%	13	7.43%	
ACO	32	58.18%	23	41.82%		47	85.45%	8	14.55%	
**Smoking (** ***n*** **, %)**										
Never	225	71.43%	90	28.57%	5.45^*^	272	86.35%	43	13.65%	5.10^*^
Yes	300	63.42%	173	36.58%		379	80.13%	94	19.87%	
**Lung function (** ***n*** **, %)**										
No report	80	54.42%	67	45.58%	42.98^***^	114	77.55%	33	22.45%	20.84^***^
FEV1 ≧ 80%	200	77.22%	59	22.78%		235	90.73%	24	9.27%	
50% ≦ FEV1 < 80%	186	71.26%	75	28.74%		212	81.23%	49	18.77%	
30% ≦ FEV1 < 50%	52	48.15%	56	51.85%		81	75.00%	27	25.00%	
FEV1 < 30%	7	53.85%	6	46.15%		9	69.23%	4	30.77%	
**DM (** ***n*** **, %)**										
No	452	68.69%	206	31.31%	7.68^*^	552	83.89%	106	16.11%	4.524^*^
Yes	73	56.15%	57	43.85%		99	76.15%	31	23.85%	
**CVD (** ***n*** **, %)**										
No	366	70.66%	152	29.34%	11.05^*^	442	85.33%	76	14.67%	7.753^*^
Yes	159	58.89%	111		41.11%	209	77.41%	61	22.59%	
**Lung cancer (** ***n*** **, %)**										
No	513	67.50%	247	32.50%	7.38^*^	634	83.42%	126	16.58%	9.694^*^
Yes	12	42.86%	16	57.14%		17	60.71%	11	39.29%	
**Eosinophil**										
Eosinophil < 2%	134	46.21%	156	53.79%	12.310^*^	208	71.72%	82	28.28%	4.060^*^
Eosinophil > 2%	151	61.38%	95	38.62%		195	79.27%	51	20.73%	
**Neutrophil**										
Neutrophil < 65%	149	60.82%	96	39.18%	10.592^*^	197	80.41%	48	19.59%	6.595^*^
Neutrophil > 65%	136	46.74%	155	53.26%		206	70.79%	85	29.21%	

AE, acute exacerbation; COPD, chronic obstructive pulmonary disease; ACO, Asthma/COPD overlapping; DM, diabetes mellitus; CVD, cardiovascular disease.

* *p* < 0.05;

** *p* < 0.01;

*** *p* < 0.001.

Patients with Eosinophil (EOS) levels <2% had a higher likelihood of experiencing a severe acute exacerbation (53.79%, *p* < 0.05) and, when compared to the number of severe acute exacerbations per year, it was found that these patients were also more likely to experience two or more severe exacerbations per year (28.28%, *p* < 0.05). Similarly, patients with Neutrophil levels > 65% had a higher likelihood of experiencing a severe acute exacerbation (53.26%, *p* < 0.05) and, when compared to the number of severe acute exacerbations per year, it was found that these patients were also more likely to experience two or more severe exacerbations per year (29.21%, *p* < 0.05). These findings suggest that during acute exacerbation or infection, there is a tendency for an increase in Neutrophil levels and a decrease in Eosinophil levels (Table 2).

**Figure 1:**
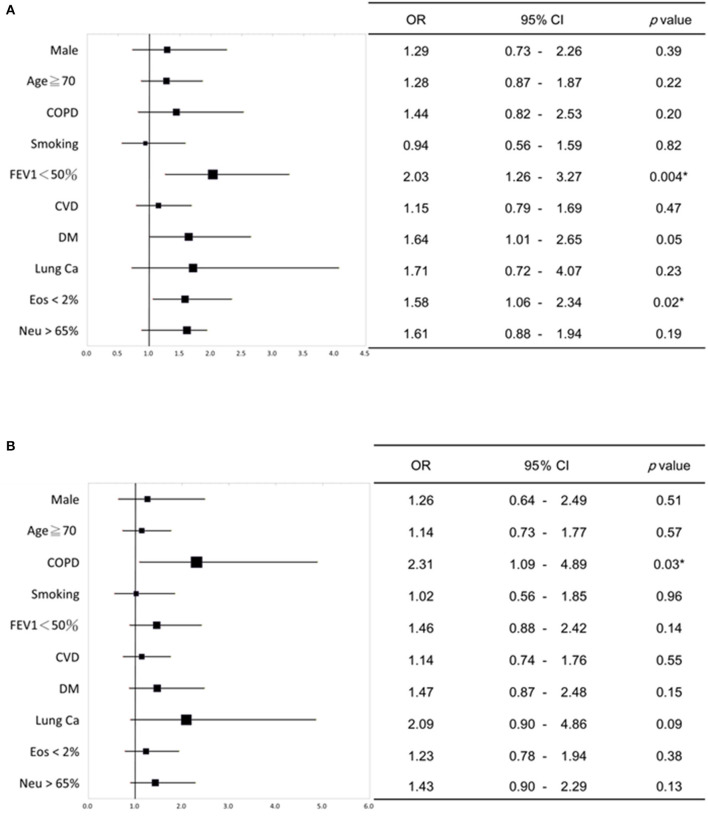
Multivariate logistic regression analysis of the relationship between intrinsic factors and severe acute exacerbation of chronic airway disease. **(A)** Patients with chronic airway disease, impaired lung function (FEV1 < 50%), diabetes, and low eosinophil levels in blood (<2%) showed a significant link to severe acute exacerbation. **(B)** Patients with obstructive lung diseases (COPD and ACO) showed a significant relationship with having two or more acute exacerbations per year. The forest plots in **(A, B)** depict the odds ratio (OR) values, while the upper and lower lines indicate the 95% confidence interval (CI). *P*-values below 0.05 were considered statistically significant. COPD, chronic obstructive pulmonary disease; FEV1, forced expiratory volume in one second; CVD, cardiovascular disease; DM, diabetes mellitus; Ca, Cancer; Eos, Eosinophil; Neu, neutrophil. *P < 0.05.

Multivariate logistic analysis was conducted to investigate the relationship between intrinsic factors (such as age, gender, diagnosis of lung disease, smoking history, comorbidities, blood data, and lung function) and the presence of severe acute exacerbation. It was found that patients with chronic airway disease and poor lung function (FEV1 < 50%), diabetes and blood data EOS < 2% were significantly related to severe acute exacerbation (Figure 1A). Patients with obstructive lung disease (COPD and ACO), have a significant correlation with experiencing two or more acute exacerbations annually (Figure 1B).

### The relationship between acute exacerbation and air pollutant

**Figure 2:**
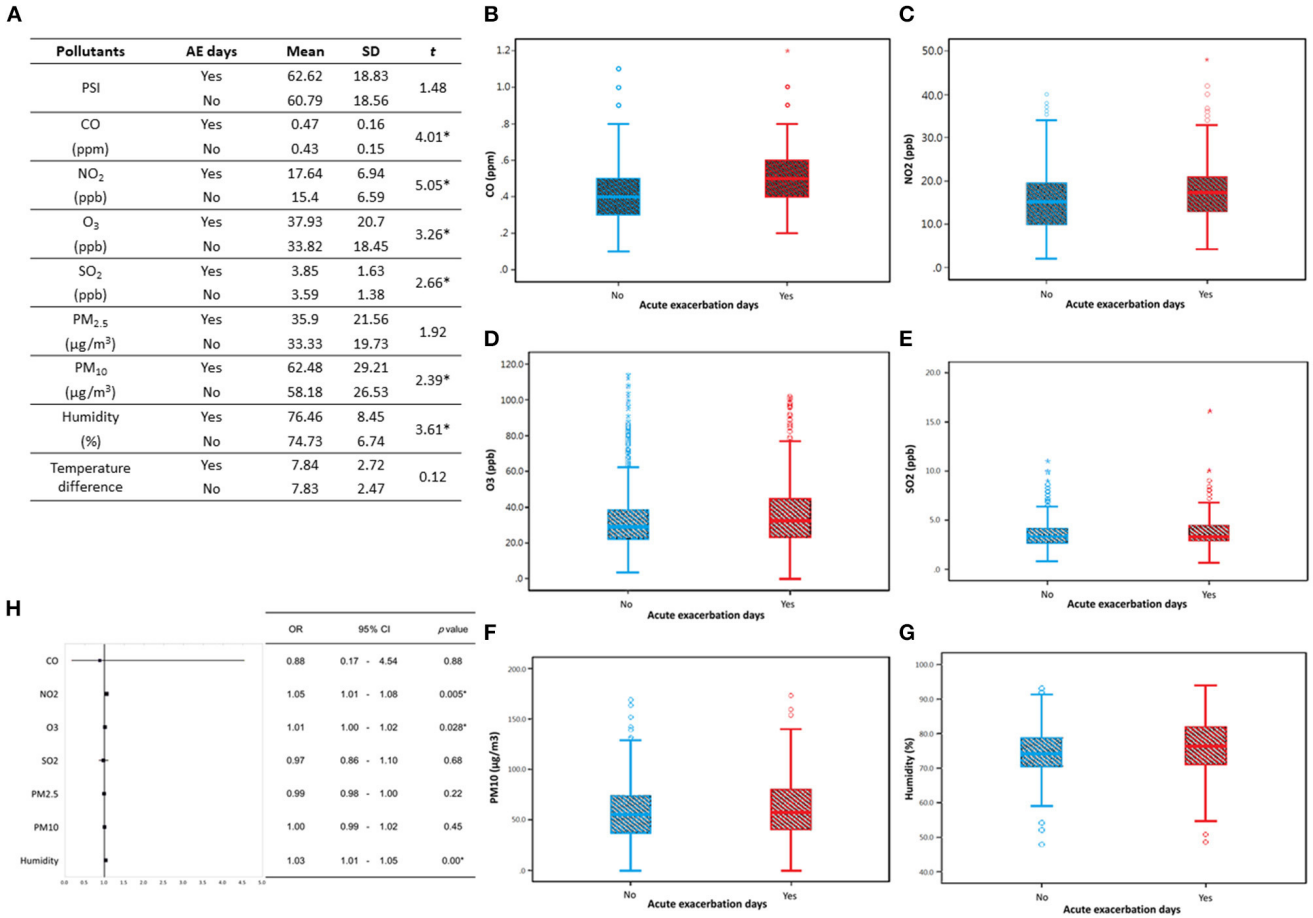
Compare the acute exacerbation days of chronic respiratory disease patients with the recorded daily pollution values. **(A)** There is a significant difference in the daily pollution values (CO, NO_2_, O_3_, SO_2_, PM_10_, humidity) on the days of acute exacerbation analyzed by independence *T*-test. Analyze the daily pollution values **(B)** CO; **(C)** NO_2_; **(D)** O_3_; **(E)** SO_2_; **(F)** PM_10_; **(G)** humidity, with or without acute exacerbation. The bar graphs indicated mean value with one standard deviation, and the box indicated the 25–75 percentile of each pollution values. These pollutants show higher levels at the days of acute exacerbation. **(H)** A multivariate logistic regression analysis combining daily pollution values revealed that the potential causes of acute exacerbation in patients with chronic respiratory disease could be NO_2_, O_3_, and humidity. The forest plots illustrate the odds ratio (OR) values, with the upper and lower lines representing the 95% confidence interval (CI). Statistical significance was determined using a threshold of *p*-values below 0.05. AE, Acute exacerbation; SD, standard deviation; PSI, pollutant standards index; OR, odds ratio; CI, confidence interval. *P < 0.05.

A comparison analysis of acute exacerbation days of chronic airway disease patients with recorded daily pollution values (CO, NO_2_, O_3_, SO_2_, PM_10_, and humidity) (Figure 2A) shows that the presence of acute exacerbation days has reached a significant level (*p* < 0.05) in the values of these pollutants, indicating that on the day of patients with acute exacerbation, there are higher levels of CO, NO_2_, O_3_, SO_2_, PM_10_, and humidity (Figures 2B–G). After performing a multivariate logistic regression combining daily pollution values, it was found that the main extrinsic factors that may cause acute exacerbation in patients with chronic airway disease are likely to be NO_2_, O_3_, and humidity (Figure 2H). Environmental factors monthly and annual trend graphs showed that the concentration of NO_2_ and O_3_ is higher in spring and winter, while the relative humidity is higher in May of each year (Supplementary Figure 1).

The analysis of the possible main causes of acute exacerbations of chronic airway disease (NO_2_, O_3_, humidity) in relation to the average monthly concentrations of 2014–2016, revealed that the concentration of NO_2_ (Supplementary Table 1) was significantly higher between November and January than in other months; the concentration of O_3_ (Supplementary Table 2) was significantly higher between September and November than in other months. This indicates that the concentrations of NO_2_ and O_3_ are significantly higher in the spring and winter seasons, which is consistent with the fact that patients with chronic airway diseases often experience severe acute exacerbations during these seasons. Humidity (Supplementary Table 3) was significantly higher between April and June, which is consistent with the fact that it is the rainy season and humidity is heavier during this period.

### Cross-analysis of patients with chronic airway disease during severe acute exacerbation

This study analyzes the dates on which patients with chronic airway diseases visited emergency or hospitalized due to acute deterioration, and examines whether certain months are more likely to cause severe acute exacerbation in patients. Although the test results are not significant, it can be seen that the proportion of patients experiencing severe acute exacerbation in spring and winter is higher than in other months (Supplementary Table 4).

**Figure 3:**
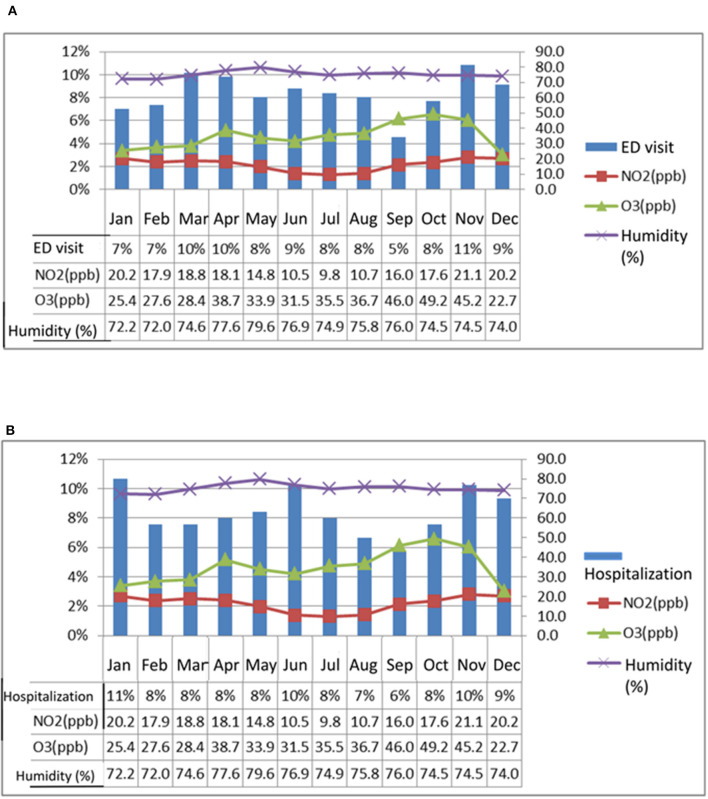
This study compares the monthly average concentrations of NO_2_, O_3_, and humidity from 2014 to 2016 with the months in which patients with chronic airway disease experienced acute exacerbation and sought treatment at the emergency department (ED) or were hospitalized. The analysis, as depicted in **(A, B)**, reveals that NO_2_ and O_3_ concentrations are higher in spring and winter, and correspondingly, a higher number of patients seek medical treatment. While statistical analysis shows that humidity is highest from April to June, the number of patients visiting the hospital during these months is not necessarily the highest, particularly in May when humidity is at its peak.

This study compares the monthly averages of NO_2_, O_3_, and humidity levels from 2014 to 2016 with the months in which patients with chronic airway diseases visited the emergency department or were hospitalized. The comparison is shown in Figures 3A, B. It is found that the levels of NO_2_ and O_3_ are higher in spring and winter, and there are also more people visiting the hospital. In terms of humidity, the previous statistical analysis stated that humidity is significantly higher in April-June than in other months, but this figure shows that May, when humidity is high, is not the month when the most people visit the hospital.

### Monthly acute exacerbation and air pollutant regression analysis

Multivariate linear regression analysis was used to examine the effects of NO_2_, O_3_, and humidity on the occurrence of severe acute exacerbations of chronic airway diseases. It was found that humidity had the greatest effect on the occurrence of severe acute exacerbations, followed by NO_2_, while O_3_ had no significant effect (Table 3). The combined explanatory power of the three variables was 71.8%. The results of the multiple regression analysis showed that for every 1 ppb increase in NO_2_, the number of severe acute exacerbations increased by 46.4%, and for every 1% increase in humidity, the number of severe acute exacerbations increased by 52.6%. Both were statistically significant, as shown in Table 3.

**Table 3:** Linear regression analysis of monthly acute exacerbation events and air pollutants NO_2_, O_3_, and humidity.

**Model**		**Non-standardized coefficient**		**Standardized coefficient**	***t***	***p***
		**B**	**Standard error**	**Beta**		
1	(Constant)	−0.428	3.019		−0.142	0.888
	NO_2_	0.591	0.177	0.496	3.334	0.002
2	(Constant)	−1.177	3.171		−0.371	0.713
	NO_2_	0.51	0.204	0.429	2.504	0.017
	O_3_	0.058	0.072	0.139	0.811	0.423
3	(Constant)	−60.81	14.776		−4.115	< 0.001
	NO_2_	0.553	0.168	0.464	3.292	0.002
	O_3_	−0.011	0.061	−0.025	−0.172	0.864
	Humidity	0.815	0.199	0.526	4.1	< 0.001

Independent variable: events of severe acute exacerbations.

**Figure 4:**
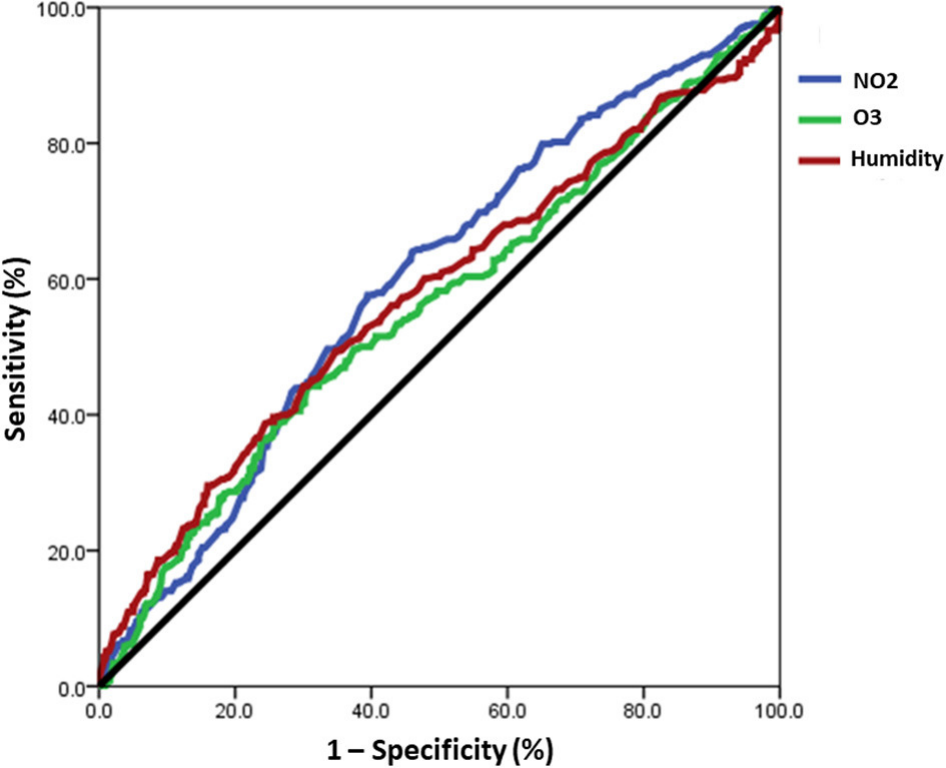
The ROC curve analysis revealed a correlation between the daily values of NO_2_, O_3_, and humidity and the occurrence of acute exacerbation days in patients with chronic airway disease. A risk of severe acute exacerbation of chronic airway disease in patients with chronic airway disease was found when the concentration of NO_2_ in the air exceeds 16.65 ppb (AUC: 0.597), O_3_ exceeds 35.65 ppb (AUC: 0.557), and humidity is above 76.95% (AUC: 0.575).

The study aimed to examine the relationship between daily pollution values of NO2, O3, and humidity, and the incidence of acute exacerbation days in patients with chronic airway disease using ROC curve analysis (Figure 4). Concentrations of NO2 in the air greater than 16.65 ppb were found to be associated with an increased risk of severe acute exacerbation [area under curve (AUC): 0.597, 95% CI: 0.560–0.633]. Similarly, O3 concentrations exceeding 35.65 ppb were linked to a higher risk of severe acute exacerbation (AUC: 0.557, 95% CI: 0.519–0.595). Additionally, humidity levels above 76.95% were associated with an elevated risk of severe acute exacerbation (AUC: 0.575, 95% CI: 0.536–0.614). The results demonstrated that exceeding specific concentration thresholds of NO_2_, O_3_, and humidity plays a crucial role in the occurrence of severe acute exacerbation in patients with chronic airway disease.

## Discussion

The findings of our study demonstrate a significant association between severe acute exacerbation and specific clinical factors in patients with chronic airway diseases. Specifically, impaired lung function (FEV1 < 50%), diabetes, and low eosinophil levels in the blood (<2%) were strongly linked to the occurrence of severe acute exacerbations. Moreover, patients diagnosed with obstructive lung diseases, such as COPD and ACO, exhibited a significant relationship with experiencing two or more acute exacerbations per year. The study also found that there are higher levels of CO, NO_2_, O_3_, SO_2_, PM_10_, and humidity on the day of acute exacerbation, and that the main causes of acute exacerbation in patients with chronic airway disease are significantly related to NO_2_, O_3_, and humidity. When the air NO_2_ > 16.65 ppb, O_3_ > 4.75 ppb, or humidity > 76.95%, there is a risk of acute exacerbation in patients with chronic airway disease.

This study found that patients over the age of 70, men, those who have previously smoked and those who currently smoke, as well as those with worse lung function (FEV1 < 50%), had a higher proportion of acute exacerbations of chronic airway disease. Therefore, quitting smoking early, elderly patients should receive regular flu and pneumonia vaccinations, and lung rehabilitation exercises and breathing exercises can effectively reduce the risk of acute exacerbation of chronic airway disease by slowing the deterioration of lung function (1).

The interest in utilizing biomarkers to identify COPD and asthma patients at risk of exacerbations is rapidly growing. An association has been observed between increased blood and sputum eosinophils and more frequent exacerbations (15). Currently, blood eosinophil counts (≥300 cells/μL) serve as a guideline for identifying COPD and asthma patients at higher risk of exacerbations, as well as those more likely to benefit from treatment with inhaled corticosteroids or biologic agents (13, 16). Patients with higher blood eosinophil counts in COPD and asthma also exhibit elevated lung eosinophil numbers and higher levels of markers indicating type-2 inflammation in the airways (17, 18). Furthermore, there is an increase in neutrophil extracellular trap formation among patients with severe COPD, which is associated with more frequent exacerbations and a loss of microbiota (19). This neutrophil extracellular trap formation is also linked to an increased exacerbation risk in patients with COPD and asthma. Our study revealed that patients with EOS levels below 2% and those with neutrophil levels above 65% are more likely to experience severe and more frequent acute exacerbations. These findings suggest that during acute exacerbation or infection, there is a tendency for an increase in neutrophil levels and a decrease in eosinophil levels. Despite the clinical heterogeneity of chronic airway disease, identifying distinct inflammatory endotypes has proven challenging, although rare genetic endotypes of COPD and asthma have been recognized. Further research is necessary to establish connections between inflammatory endotypes and clinical manifestations and outcomes in chronic airway disease, particularly in predicting the response to precision medicines.

The development of lung cancer may be related to smoking mainly. However, air pollution has been linked to the development of lung cancer. Long-term exposure to air pollution, particularly fine particulate matter (PM_2.5_) and nitrogen oxides (NOx), has been associated with an increased risk of lung cancer as found in studies (20, 21). Studies have also revealed a positive association between exposure to ambient air pollution and lung cancer incidence and mortality (22, 23). The World Health Organization's International Agency for Research on Cancer (IARC) has classified outdoor air pollution as a carcinogen to humans (24). In Taiwan, more than 50% of patients with lung cancer had never smoked. PM_2.5_ level changes can affect lung cancer incidence and patient survival (25). Therefore, high-risk patients need to regularly follow up with low-dose computed tomography screening for lung cancer. This study found a relationship between comorbidities and acute exacerbations, with lung cancer being the most common comorbidity of chronic airway disease. Meanwhile, cardiovascular disease and diabetes should be controlled through medication and lifestyle improvements, which can also effectively control chronic airway diseases.

Air pollution is a major cause of airway and allergic diseases such as asthma and COPD. Traffic and domestic fires using biomass fuels are significant sources of air pollution. Despite the challenge of measuring personal exposure to pollutants, new methods are revealing links between air pollution and airway diseases (26). COPD patients, who are often former or current smokers, are particularly sensitive to air pollution and should be protected from high levels of particulate matter (27). Studies have shown particulate matter air pollution to be a key factor in the development and exacerbation of COPD and asthma (28, 29). In addition, increased levels of NO_2_, CO, O_3_, and PM_10_ and fluctuating temperature were linked to acute COPD exacerbation in older patients (30). PM_2.5_, PM_10_, NO_2_, SO_2_, CO, O_3_, average temperature, and diurnal temperature range were found to impact COPD exacerbations among various air pollutants and meteorological factors (31).

Exacerbations are primarily instigated by respiratory viral infections; however, bacterial infections and environmental factors, including ambient air pollution and elevated temperatures, can also contribute to initiating and amplifying these events (32, 33). Short-term exposure to fine particulate matter (PM_2.5_) and coarse particulate matter (PM_10_) is linked to escalated rates of hospitalizations, ED visits, outpatient consultations (33), and heightened mortality in cases of COPD exacerbations (32, 34, 35). A growing body of research indicates the association between air pollution and acute exacerbation of chronic obstructive pulmonary disease (AECOPD) (36–38). Our study results revealed that the highest number of hospitalizations occurred during the first month when both NO2 and O3 levels were at their lowest. Similar patterns were observed in March and April for ED visits. Exposure to high levels of PM_2.5_, PM_10_, and SO_2_, as well as low levels of NO_2_ and high levels of CO, were found to increase the risk of AECOPD. The cumulative exposure-response curves showed different trends: approximately linear for PM_2.5_, “V”-shaped for PM_10_, “U”-shaped for NO_2_, and inverted-“V” for SO_2_, CO, and O_3_. Specifically, high levels of SO_2_, NO_2_, and extreme concentrations of PM_2.5_ had the most pronounced impact on the day of exposure, and these effects persisted for a certain duration. This suggests that the immediate effects of these air pollutants were stronger than the delayed effects. Furthermore, low levels of SO_2_ and CO exhibited a protective effect on AECOPD, which gradually increased over time until a lag of 27 days (39). The nonlinear effects of different air pollutants on AECOPD varied based on factors such as gender, age, and seasons (40).

According to the study results, it was found that there is a relationship between NO_2_, O_3_, humidity, and acute exacerbation of chronic airway diseases, but no obvious connection with suspended particulate matter PM_2.5_ and PM_10_. Therefore, the exacerbation of chronic airway diseases may be caused by pollutants carried by suspended particulate matter. However, current air pollution prevention laws do not have regulations on the concentration limits of these pollutants. In the future, it is hoped that further research will confirm the harmful effects of certain concentrations of NO_2_ and O_3_ on human health, leading to legislation that monitors pollution sources and reduces emissions. Taiwan has an island climate with high humidity. However, it remains unknown if the proportion of acute exacerbation of chronic airway diseases in Taiwan is higher than in countries with a continental climate. The study results showed that humidity is linked to acute exacerbation of chronic airway diseases and a humid environment can promote the growth of dust mites, mosquitoes, or fungi. Therefore, it is recommended that patients with chronic airway diseases use dehumidifiers and other equipment to reduce the humidity in their homes.

The study has some limitations. It only sampled patients with chronic airway diseases from a single medical center in Taiwan between 2015 and 2016, with insufficient sample size and reference time, and may not reflect patient behavior or regional differences. The study didn't consider patients who sought treatment elsewhere during acute episodes and didn't examine physician medication choices or patient medication adherence. In the future, the study aims to obtain more data via the National Health Insurance database.

## Conclusions

This study found that patients over the age of 70, who are male, smokers, and have worse lung function, had a higher proportion of acute exacerbations of chronic airway disease. Additionally, the study found that patients with certain blood cell counts and those exposed to certain air pollutants had a higher likelihood of experiencing severe acute exacerbations. The study suggests that reducing exposure to these pollutants and encouraging quitting smoking could help lower the risk of acute exacerbations. The findings of this study may be useful in identifying patients at high risk for severe acute exacerbations and in developing targeted interventions to prevent exacerbations. Reduced air pollution from industrial upgrades, vehicle and fuel renovations, better public transportation and healthy city development can prevent respiratory diseases. A comprehensive national environmental policy is needed to address this pressing issue (24). A warning system, such as a mobile app, should be created to help patients with chronic respiratory diseases prepare and take preventive measures in advance.

## Data availability statement

The original contributions presented in the study are included in the article/Supplementary material, further inquiries can be directed to the corresponding author.

## Ethics statement

The studies involving humans were approved by the Research Ethics Committee of National Taiwan University Hospital and National Taiwan University Hospital Yunlin Branch (201411019RINB). The studies were conducted in accordance with the local legislation and institutional requirements. The ethics committee/institutional review board waived the requirement of written informed consent for participation from the participants or the participants' legal guardians/next of kin because this is a retrospective study.

## Author contributions

C-HC and Y-FC wrote the main manuscript text. H-CP prepared figures. C-YC revised the manuscript. B-WC supervised the study. All authors reviewed the manuscript. All authors contributed to the article and approved the submitted version.
