# Supplementary material for: Environmental pollutants increase the risks of acute exacerbation in patients with chronic airway disease
Source: Front Public Health. 2023 Oct 30;11:1215224. doi: 10.3389/fpubh.2023.1215224 (PMC10643209; doi:10.3389/fpubh.2023.1215224)

Supplementary Table 1： ANOVA analysis of the monthly concentration changes of the environmental pollutant NO_2

	Month	Mean ± SD			*f*	*p*	Post-Hoc
NO_2	Jan (1)	20.199	±	4.2864	47.956	< 0.001	11 = 12 = 1 = 3 > 5 = 9 > 8 = 6 = 7
	Feb (2)	17.869	±	4.5553			
	Mar (3)	18.825	±	4.0613			
	Apr (4)	17.945	±	6.7186			
	May (5)	14.804	±	6.7305			
	Jun (6)	10.451	±	3.6066			
	Jul (7)	9.874	±	4.1627			
	Aug (8)	10.703	±	5.1333			
	Sep (9)	13.859	±	6.6834			
	Oct (10)	17.690	±	7.8679			
	Nov (11)	21.145	±	6.8250			
	Dec (12)	20.154	±	3.8691			

SD: standard deviation

Supplementary Table 2: ANOVA analysis of the monthly concentration changes of the environmental pollutant O_3

	Month	Mean ± SD			*f*	*p*	Post-Hoc
O_3	Jan (1)	25.495	±	8.1794	21.42	< 0.001	4 = 9 = 10 = 11 > 8 = 7 > 12
	Feb (2)	27.632	±	8.5727			
	Mar (3)	28.467	±	9.2863			
	Apr (4)	38.485	±	19.4779			
	May (5)	33.956	±	16.8066			
	Jun (6)	31.578	±	13.8104			
	Jul (7)	35.532	±	18.5066			
	Aug (8)	36.720	±	18.1860			
	Sep (9)	45.914	±	28.4640			
	Oct (10)	49.185	±	22.6647			
	Nov (11)	45.264	±	24.1317			
	Dec (12)	22.787	±	5.9811			

SD: standard deviation

Supplementary Table 3: ANOVA analysis of the monthly concentration changes of the environmental humidity

Month		Mean ± SD			*f*	*p*	Post-Hoc
Humidity	Jan (1)	72.191	±	9.3960	8.272	< 0.001	4 = 5 = 6 > 1 = 2
	Feb (2)	72.046	±	7.4949			
	Mar (3)	74.662	±	7.9867			
	Apr (4)	77.477	±	7.1685			
	May (5)	79.635	±	7.6074			
	Jun (6)	76.928	±	6.6459			
	Jul (7)	74.924	±	8.2426			
	Aug (8)	75.878	±	6.0105			
	Sep (9)	76.033	±	5.2521			
	Oct (10)	74.585	±	5.4640			
	Nov (11)	74.572	±	5.7941			
	Dec (12)	74.084	±	6.7112			

SD: standard deviation

Supplementary Table 4. Analysis of the correlation between the month and event of emergency visits and hospitalization in acute exacerbation of patients with chronic airway disease.

Month	Emergency department visit				X^2	Hospitalization				X^2
	No		Yes			No		Yes		
Jan	74	9.14%	20	7.02%	14.338 p=0.215	70	8.05%	24	10.67%	8.28 p=0.688
Feb	64	7.90%	21	7.37%		68	7.82%	17	7.56%	
Mar	64	7.90%	29	10.18%		76	8.74%	17	7.56%	
Apr	60	7.41%	28	9.82%		70	8.05%	18	8.00%	
May	70	8.64%	23	8.07%		74	8.51%	19	8.44%	
Jun	65	8.02%	25	8.77%		67	7.70%	23	10.22%	
Jul	69	8.52%	24	8.42%		75	8.62%	18	8.00%	
Aug	70	8.64%	23	8.07%		78	8.97%	15	6.67%	
Sep	77	9.51%	13	4.56%		77	8.85%	13	5.78%	
Oct	71	8.77%	22	7.72%		70	8.05%	24	10.67%	
Nov	59	7.28%	31	10.88%		68	7.82%	17	7.56%	

Supplementary Figure 1. . Seasonal Trends in Environmental Pollution of NO2, O3 and Relative Humidity. The study analyzed monthly and annual trend graphs of environmental factors from 2014 to 2016 and found that the concentration of NO2 (A) and O3 (B) was higher in spring and winter. In contrast, the relative humidity (C) was higher in May of each year.

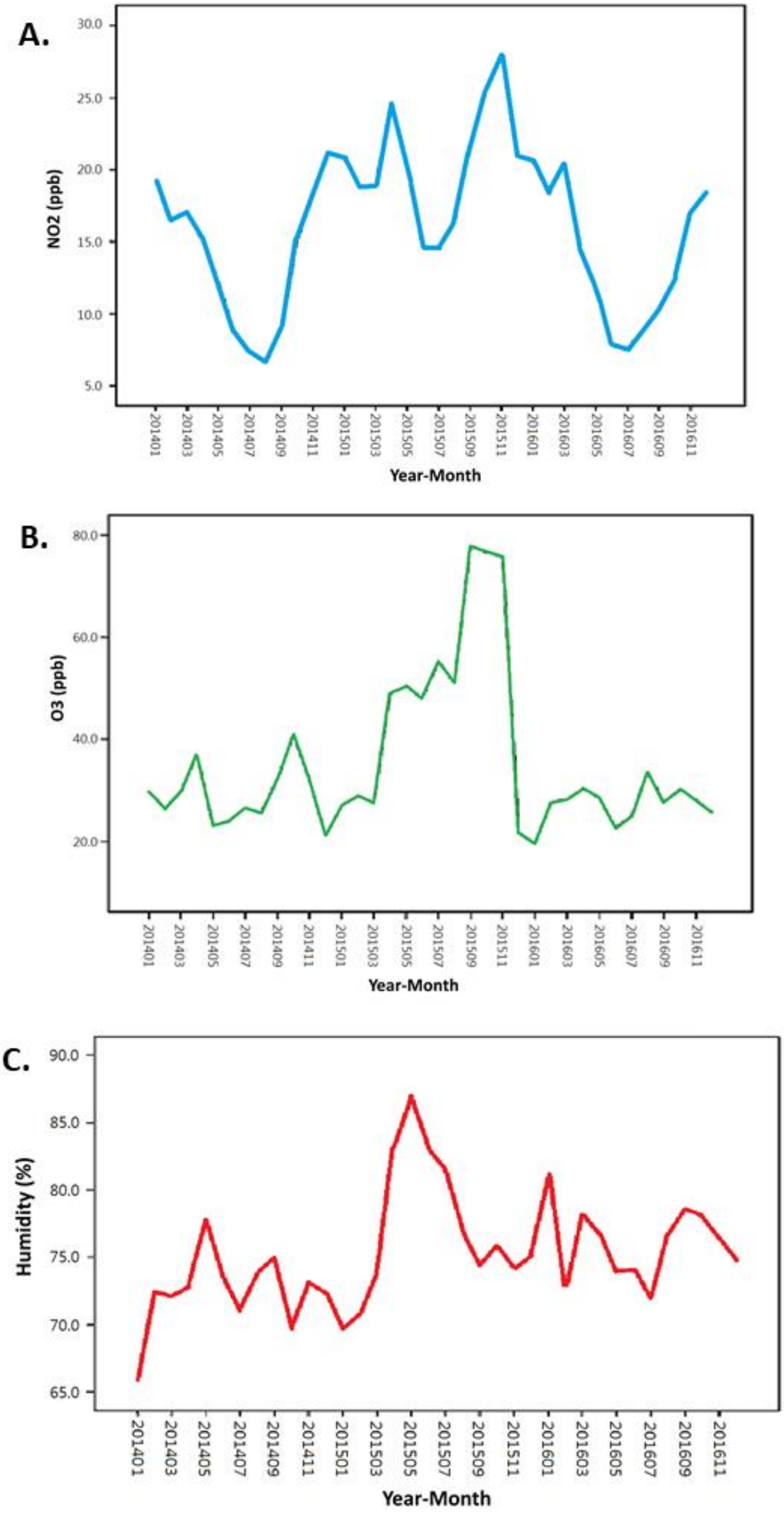

Supplement: Supplementary file 1 [file Data_Sheet_1.pdf]
